# Plant community responses to increased precipitation and belowground litter addition: Evidence from a 5‐year semiarid grassland experiment

**DOI:** 10.1002/ece3.4012

**Published:** 2018-04-14

**Authors:** Hongxia Chen, Linna Ma, Xiaoping Xin, Junyao Liu, Renzhong Wang

**Affiliations:** ^1^ State Key Laboratory of Vegetation and Environmental Change Institute of Botany The Chinese Academy of Sciences Beijing China; ^2^ University of Chinese Academy of Sciences Beijing China; ^3^ Institute of Agricultural Resources and Regional Planning Chinese Academy of Agriculture Sciences Beijing China

**Keywords:** climatic changes, community composition, community structure, plant functional group, species richness

## Abstract

Global climate change is predicted to stimulate primary production and consequently increases litter inputs. Changing precipitation regimes together with enhanced litter inputs may affect plant community composition and structure, with consequent influence on diversity and ecosystem functioning. Responses of plant community to increased precipitation and belowground litter addition were examined lasting 5 years in a semiarid temperate grassland of northeastern China. Increased precipitation enhanced community species richness and abundance of annuals by 16.8% and 44%, but litter addition suppressed them by 25% and 54.5% after 5 years, respectively. During the study period, perennial rhizome grasses and forbs had consistent negative relationship under ambient plots, whereas positive relationship between the two functional groups was found under litter addition plots after 5 years. In addition, increased precipitation and litter addition showed significant interaction on community composition, because litter addition significantly increased biomass and abundance of rhizome grasses under increased precipitation plots but had no effect under ambient precipitation levels. Our findings emphasize the importance of water availability in modulating the responses of plants community to potentially enhanced litter inputs in the semiarid temperate grassland.

## 1 INTRODUCTION

Precipitation is a major environmental factor in determining ecosystem structure and function, especially in water‐limited temperate grasslands (Burke, Lauenroth, & Parton, 1997; Epstein, Burke, & Lauenroth, 2002; Zhou, Talley, & Luo, 2009), which account for about 45% of the global land surface (Saco, Willgoose, & Hancock, 2006). Global climate models predict that mean annual precipitation (MAP) will enhance by 30–100 mm in this century at most of the midlatitude regions (IPCC, 2013; Ni & Zhang, 2000). Empirical studies have indicated that changes in precipitation regime and their direct influence on soil water availability are a dominant factor for structuring regional and local plant community structure and composition (Bai, Han, Wu, Chen, & Li, 2004; Knapp & Smith, 2001; Morecroft et al., 2004; Weltzin, Bridgham, Pastor, Chen, & Calvin, 2003). The changes in the plant biomass and abundance with changes in rooting depths and plant height would affect interspecific competition and regulate the responses of plant community to changing precipitation regimes (Yang et al., 2011).

Litter quality and quantity are important and complex factors in regulating plant productivity and richness in terrestrial ecosystems (Letts, Lamb, Mischkolz, & Romo, 2015; Weltzin et al., 2005). Anticipated global warming and enhancing CO_2_ concentration are assumed to increase plant biomass, especially belowground biomass in semiarid grassland ecosystems (IPCC, 2013; Jastrow et al., 2005), and subsequently, much more litter above‐ and belowground will be incorporated into soils. Therefore, a great deal of effort involving manipulative experiments, laboratory incubation, large‐scale observations, and meta‐analyses has been devoted to surveying plant community productivity and diversity responses to increased litter inputs following the expansion of primary production (Ma et al., 2013; Wang, Zhao, Walter, Wang, & Han, 2010; Wang et al., 2015; Weltzin et al., 2005; Xiong & Nilsson, 1999). The extra litter inputs (and the associated litter nutrient release) are considered an environmental problem that disrupts plant interspecific competition (Wang et al., 2010); but, from a management perspective, enhanced litter inputs to the soils have also been put forward as a means to reverse the loss of species diversity and promote the restoration of degraded of lands because soil fertility is mainly kept by cycling of litter nutrient in the temperate grasslands (Liu, Huang, Han, Sun, & Zhou, 2006).

Concurrent increased precipitation and enhanced litter inputs may contribute complex interactive influences on ecosystem structure and functioning. Although several studies have showed increased precipitation significantly enhanced the ratio of grass to forb biomass under litter addition, but it had no effect under ambient litter levels in temperate grasslands (Ma, Guo, Xin, Yuan, & Wang, 2013; Ma, Huang, Guo, Wang, & Xiao, 2012; Yang et al., 2011), detailed mechanistic studies evaluating the extent of which plant responses to enhanced litter inputs may be amplified or canceled by increased precipitation have largely been overlooked in a long‐term experimental investigations.

To examine the effects of increased precipitation and litter addition on plant community dynamics, we conducted a field experiment in which we increased the precipitations by setting iron boxes beside plots (0, +30%) and artificially added particulate organic matter to topsoil (0, +30%) in a temperate steppe of northeastern China. We hypothesized that (1) increased precipitation and litter addition would significantly stimulate plant richness, abundance, and biomass of different functional groups, because water and nutrient are key factors limiting productivity in temperate grassland ecosystem (Harpole, Potts, & Suding, 2007); (2) there would be additive or synergistic effects of combined increased precipitation and litter addition on plant community composition based on the above prediction.

## 2 MATERIALS AND METHODS

### 2.1 Study site

This research was conducted at the Hulunber Grassland Ecosystem Observation and Research Station of Chinese Academy of Agriculture Sciences, which is situated at Xiertala farm, the center of Hulunber steppe (49°19′N, 120°03′E, 628 m a.s.l), Inner Mongolia, China. This station is a multiyear fenced scientific observation site for a *Stipa baicalensis*‐dominated temperate meadow steppe. The annual precipitation is 350 mm, with about 90% occurring in the growing season from May to September. Mean annual air temperature is −3 to 1°C. The soil in this area is classified as dark chestnut soil according to Chinese classification or FAO classification. Soil bulk density is 1.13 g/cm^3^ and pH is 7.2 on average. The native vegetation is dominated by perennial species *S*. *baicalensis* and *Leymus chinensis* (Trin.) Tzvel. Other abundant plant species mainly include *Artemisia frigida Willd*., *Pulsatilla chinensis*,* Artemisia tanacetifolia Linn*., and *Serratula centauroides*. Total vegetation cover ranges from 60% to 75%, with an average height of the canopy of 50 cm and an average depth of the root of 30 cm (Table 1). For the information of soil organic C, total N, and inorganic N content (see Ma et al., 2012).

**Table 1:** Results (*F*‐values) of repeated measures ANOVAs on the effects of increased precipitation (P), belowground litter addition (L), year (Y), and their interactions on soil moisture (SM), plant species richness (PSR), abundance of perennial rhizome grasses (A‐PRG), perennial bunchgrasses (A‐PBG), perennial forbs (A‐PF), annuals (A‐AS), and semishrubs (A‐SS), and biomass of perennial rhizome grasses (B‐PRG), perennial bunchgrasses (B‐PBG), perennial forbs (B‐PF), annuals (B‐AS), and semishrubs (B‐SS)

	SM	PSR	A‐PRG	A‐PBG	A‐PF	A‐AS	A‐SS	B‐PRG	B‐PBG	B‐PF	B‐AS	B‐SS
P	384.33**	47.54***	3.05	0.04	3.37	7.59**	2.91	2.07	2.46	2.34	3.04	0.57
L	54.53***	79.47***	2.10	1.47	1.16	5.00*	1.57	1.77	2.58	3.02	1.95	3.12
P × L	67.05***	4.97*	283.97***	3.42*	2.83	26.52***	1.78	105.95***	2.73	0.21	0.71	1.42
Y	43.22***	247.74***	89.82***	119.40***	331.16***	134.40***	37.86***	194.42***	28.74***	19.35***	4.52*	7.74**
Y × P	47.22***	22.94***	4.76*	2.43	4.21*	26.32***	5.32*	2.65	4.77*	3.70	4.96*	1.80
Y × L	34.70***	36.89***	2.57	2.56	3.20	26.74***	4.18*	2.53	4.73*	2.18	2.76	4.17*
Y × P × L	3.04*	1.46	3.87*	19.17***	5.49*	9.65***	4.05*	16.36***	1.06	2.53	3.02	1.26

*, **, and *** represent significant at *p* < .05, .01, and .001, respectively.

### 2.2 Experimental setup

The experimental site with uniform vegetation was selected in May 2010. The site has been fenced since 1997 to prevent grazing by large vertebrate herbivores. There were 24 2 × 2 m plots included in this experimental area, the establishment of which was applied in a randomized complete block experimental design. Two‐meter spacing existed between the adjacent plots. The experiment had four treatments with six replicates each.

Increased precipitation treatment simulating a 30% precipitation increase in growing season from 2010 to 2014 was based on climate models which predict that MAP will increase by 30–100 mm in this century in the semiarid temperate grassland (Ni & Zhang, 2000). Outside each increased precipitation plot, there were two identical open‐top iron boxes (length 85 cm, width 71.5 cm, and height 15 cm) (Appendix S1). The base area of each iron box was approximately 15% of each increased precipitation plot (2 × 2 m). First, a circular hole, whose inner diameter was 1.5 cm, was punched on one side of the box (facing the plot) and then a rubber water pipe was connected to the hole. Therefore, the rains falling into the boxes quickly flowed to the plots through these water pipes. Each pipe was perforated and formed a series of small holes on it and then was arranged an S‐shaped on the ground so that the additional rainfall flowed evenly into the plots.

Senescent plant was collected from an adjacent field and then air‐dried and milled to <2 mm length before use. Litter added to 0–10 cm surface soil layer was at rates equivalent to 0 and 360 g/m^2^. Because the ecosystem total plant above‐ and belowground biomass was about 1,200 g m^−2^ **year^−1^ (Ma et al., 2012), the amount of these litter addition corresponds to increases in ecosystem production of 0% and 30%. The addition rate was planned to approximate projected increases in NPP of temperate steppes by 26%–61% under the circumstances of CO_2_ concentration doubling (Gao & Yu, 1998). The C, N, and P contents, C: N ratio, and lignin content of the soil organic matter were 40.3%, 0.3%, 0.03%, 144.6%, and 20.4%, respectively. We expected to add the particulate litter to the 0–10 cm soil layers without intensely damaging the root systems, thus we carefully loosened the surface soil (0–10 cm) with sharp forks, and added a predetermined quantity of particulate litter to the soil in the 0–10 cm layer homogeneously and gradually. The soil pores were carefully filled with soil and gently compacted by hand. To keep consistent soil disturbance across treatments, the plots with no litter addition were processed in the same methods as the plots that received litter addition (Ma et al., 2012, 2013).

### 2.3 Measurement

A permanent 1 × 1 m quadrat was set at the center of each plot, and we measured yearly plant community richness, abundance, and biomass responses to increased precipitation and belowground litter addition at the peak of plant biomass in early August from 2010 to 2014. All plant species were divided into five functional groups based on life form: perennial rhizome grasses (PRG), perennial bunchgrasses (PBG), perennial forbs (PF), annuals (AS), and semishrubs (SS) (Appendix S2). The presence of species in the measured quadrats was recorded as species richness of the plant community (Yang et al., 2011). By counting the occurrence of species in the 100 grids, community composition was quantified. We used individual species frequency to represent the abundance of the species (Klanderud & Totland, 2005).

We conducted a nondestructive way by establishing regression equations to estimate peak biomass of plant functional groups in this study. In order to include all the species occurred in our study area, 15 random calibration plots (1 × 1 m) just near our experimental plots were selected. We used the mean values of at least four random measurements of the species height to represent the plant height of each species in one plot. We also noted the specie frequency of each species (Yang et al., 2011). We developed regression equations among peak biomass and specie frequency and plant height for each species for the calibration plots. There were good correlations in all species among peak biomass and specie frequency and plant height in the 5 years. Finally, we used the regression equations to estimate the peak biomass of each species in the four treatments plots. The biomass of the five functional groups in each plot was the sum of biomass of each species, respectively.

### 2.4 Statistical analysis

Seasonal mean values of soil moisture used in this study were calculated from the monthly mean values, which were first averaged from all measurements in the same month. Repeated measures ANOVAs were used to examine the temporal (interannual) variations and the effects of increased precipitation and belowground litter addition on plant species richness, abundance, and biomass of functional groups. Between‐subject effects were evaluated as increased precipitation, belowground litter addition, and their interactions, and within‐subject effects were year and its interactions with increased precipitation or belowground litter addition. Stepwise multiple linear analyses were used to determine the relationships of soil moisture and temperature, soil organic C, total N, and inorganic N with plant community variables. Constrained ordination model–redundancy analyses (RDA) were conducted with soil moisture, four treatments as explanatory variables, and species richness, abundance, and biomass of the five functional groups as response variables. The response variables were log‐transformed (X’ = log_10_10 × X + 1), centered, and standardized to zero mean. Correlation analyses were used to determine the relationships among soil moisture, plant species richness, abundance, and biomass of functional groups. Increased precipitation effects on plant variables were calculated as [100 × (*P* − *A*)/*A*] in the un‐litter addition plots and [100 × (PL − *L*)/*L*] in the litter addition plots. Litter addition effects were calculated as [100 × (*L* − *A*)/*A*] in the un‐increased precipitation plots and [100 × (PL − *P*)/*P*] in the increased precipitation plots. *A*,* P*,* L*, and WN refer to ambient, increased precipitation, litter addition, and combined increased precipitation and litter addition, respectively. Increased precipitation and litter addition effects were calculated in 2014. Statistical analyses were conducted using SPSS (SPSS 21.0 for Windows, USA) and Canoco (Canoco 4.5 package, USA).

## 3 RESULTS

### 3.1 Precipitation and soil microclimate

**Figure 1:**
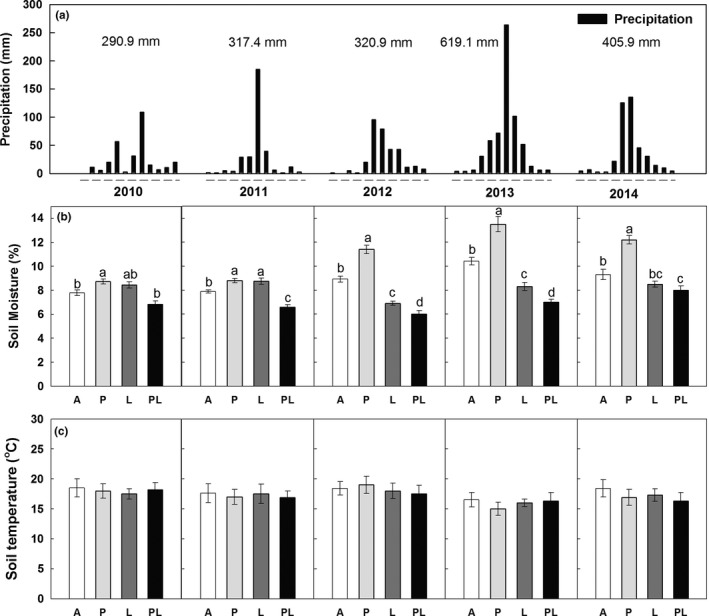
Monthly precipitation (bars) from 2010 to 2014 (a). Effects of increased precipitation and belowground litter addition on soil moisture (averaged from 2010 to 2014) (b) and soil temperature (c) at the depth of 10 cm in the semiarid temperate grassland of northeast China. Vertical bars indicate standard errors of means (*n* = 6). Difference lowercase letters indicate statistically significant differences (*p *<* *.05). *A* = ambient condition (control), *P* = increased precipitation, *L* = belowground litter addition, and PL = increased precipitation and belowground litter addition

Total precipitations over the entire year in 2010 (290.9 mm), 2011 (317.4 mm), and 2012 (320.9 mm) were 16.8%, 9.4%, and 8.3% lower than the long‐term MAP (350 mm), whereas in 2013 (619.1 mm) and 2014 (405.9 mm) were 76.8% and 15.9% higher than MAP, respectively (Figure 1a). During the 5 years, increased precipitation increased soil moisture, on average, by 12%, 11%, 27.8%, 29.6%, and 31% in 2010, 2011, 2012, 2013, and 2014 (*p *<* *.05), while belowground litter addition showed no effects on soil moisture in the first 2 years and decreased soil moisture by 22.6%, 20.3%, and 8.6% in 2012, 2013, and 2014, respectively (Figure 1b; Table 1). There was a significant interaction between increased precipitation and litter addition in affecting soil moisture (*p *<* *.001; Table 1), in that increased precipitation significantly increased soil moisture under ambient plots but not under litter addition plots. However, there were no significant effects of increased precipitation and litter addition on soil temperature across the 5 years (Figure 1c).

### 3.2 Plant species richness

**Figure 2:**
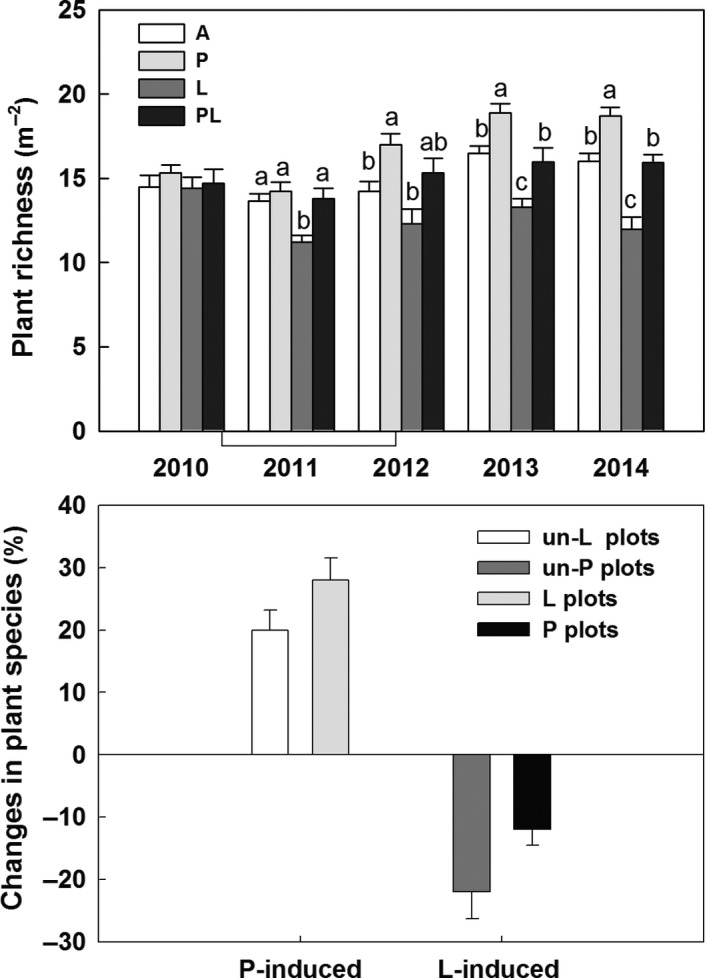
Plant species richness in responses to increased precipitation and belowground litter addition across the 5 years (2010–2014) (a). Increased precipitation induced changes in species richness in the un‐litter addition plots and litter addition plots, and litter addition induced changes in richness in the un‐increased precipitation plots and increased precipitation plots after 5 years in the temperate grassland of northeast China (b). Vertical bars indicate standard errors of means (*n* = 6). Difference lowercase letters indicate statistically significant differences (*p *<* *.05). A = ambient condition (control), *P* = increased precipitation, *L* = belowground litter addition, PL = increased precipitation and belowground litter addition; un‐L = un‐litter addition, and un‐P = un‐increased precipitation

The effects of increased precipitation and litter addition on plant species richness significantly varied with year (Table 1). Increased precipitation increased species richness, on average, by 19.4%, 14.5%, and 16.8% in 2012, 2013, and 2014, but belowground litter addition suppressed it by 18.6%, 19.4%, and 25% in 2011, 2013, and 2014, respectively (Figure 2a). There was no interaction between increased precipitation and litter addition on plant species richness across the 5 years (Table 1). The increased precipitation induced enhancement of species richness was similar in the un‐litter addition plots and litter addition plots, and litter addition induced decrease in species richness was also similar in the un‐increased precipitation plots and increased precipitation plots after 5 years (Figure 2b).

### 3.3 Plant functional group abundance

**Figure 3:**
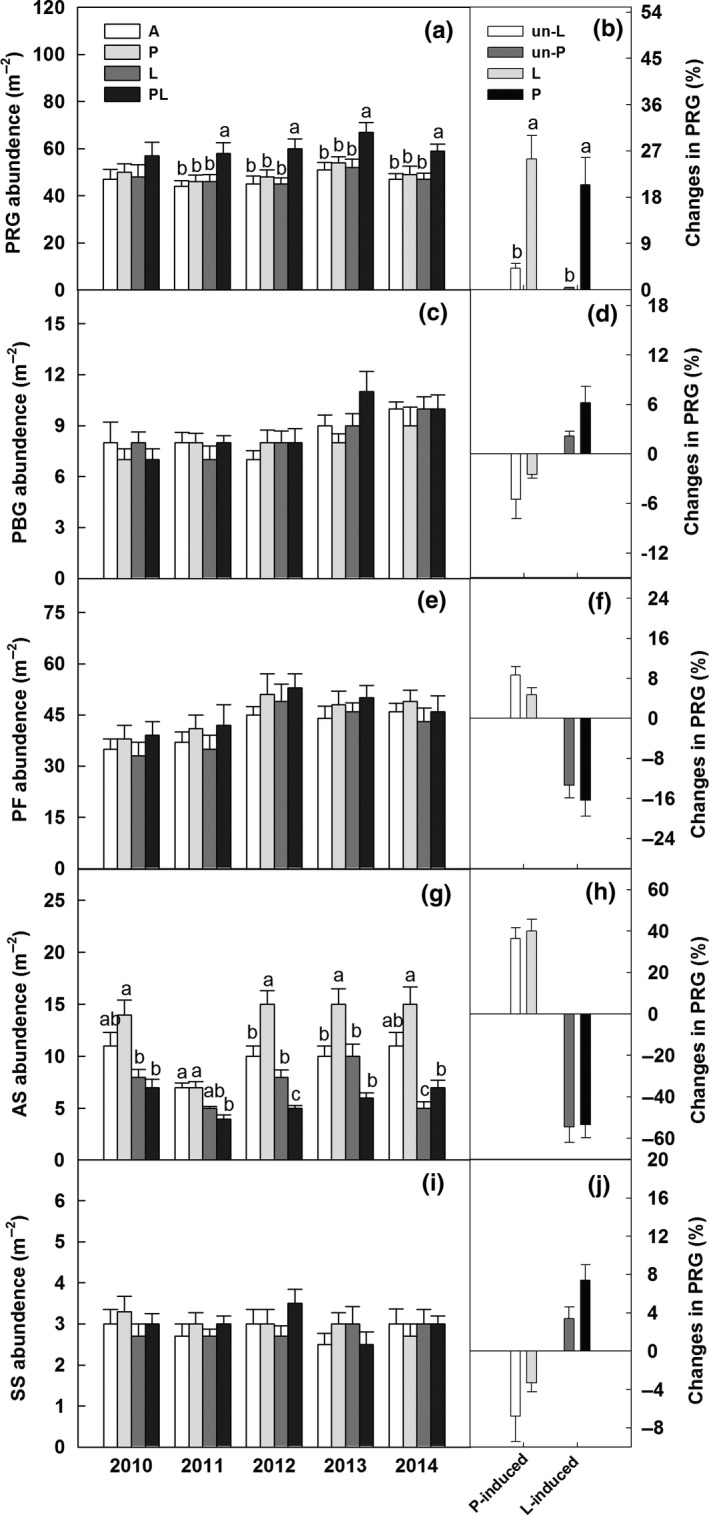
Effects of increased precipitation and belowground litter addition on abundance of perennial rhizome grasses (PRG), perennial bunchgrasses (PBG), perennial forbs (PF), annuals (AS), and semishrubs (SS) from 2010 to 2014 (a, c, e, g, i). Increased precipitation induced changes in abundance of five functional groups in the un‐litter addition plots and litter addition plots, and litter addition induced changes in abundance of five functional groups in the un‐increased precipitation plots and increased precipitation plots after 5 years in the temperate grassland of northeast China (b, d, f, h, j). Vertical bars indicate standard errors of means (*n* = 6). Difference lowercase letters indicate statistically significant differences (*p *<* *.05). *A* = ambient condition (control), *P* = increased precipitation, *L* = belowground litter addition, PL = increased precipitation and belowground litter addition; un‐L = un‐litter addition, and un‐P = un‐increased precipitation

The effects of increased precipitation and belowground litter addition on abundance changed with year (Table 1). Increased precipitation significantly enhanced abundance of annuals by 50%, 50%, and 44% in 2012, 2013, and 2014, and litter addition decreased it by 54.5% in 2014, respectively (Figure 3a,c,e,g,i). However, abundance of perennial rhizome and bunchgrasses and semishrubs did not change under increased precipitation or litter addition plots across the 5 years. Significant interactive effect of increased precipitation and litter addition on abundance of PRG was detected (Table 1). The litter addition induced enhancement of abundance of PRG was significantly lower in the un‐increased precipitation than increased precipitation plots after 5 years (Figure 3b,d,f,h,j).

### 3.4 Plant functional group biomass

**Figure 4:**
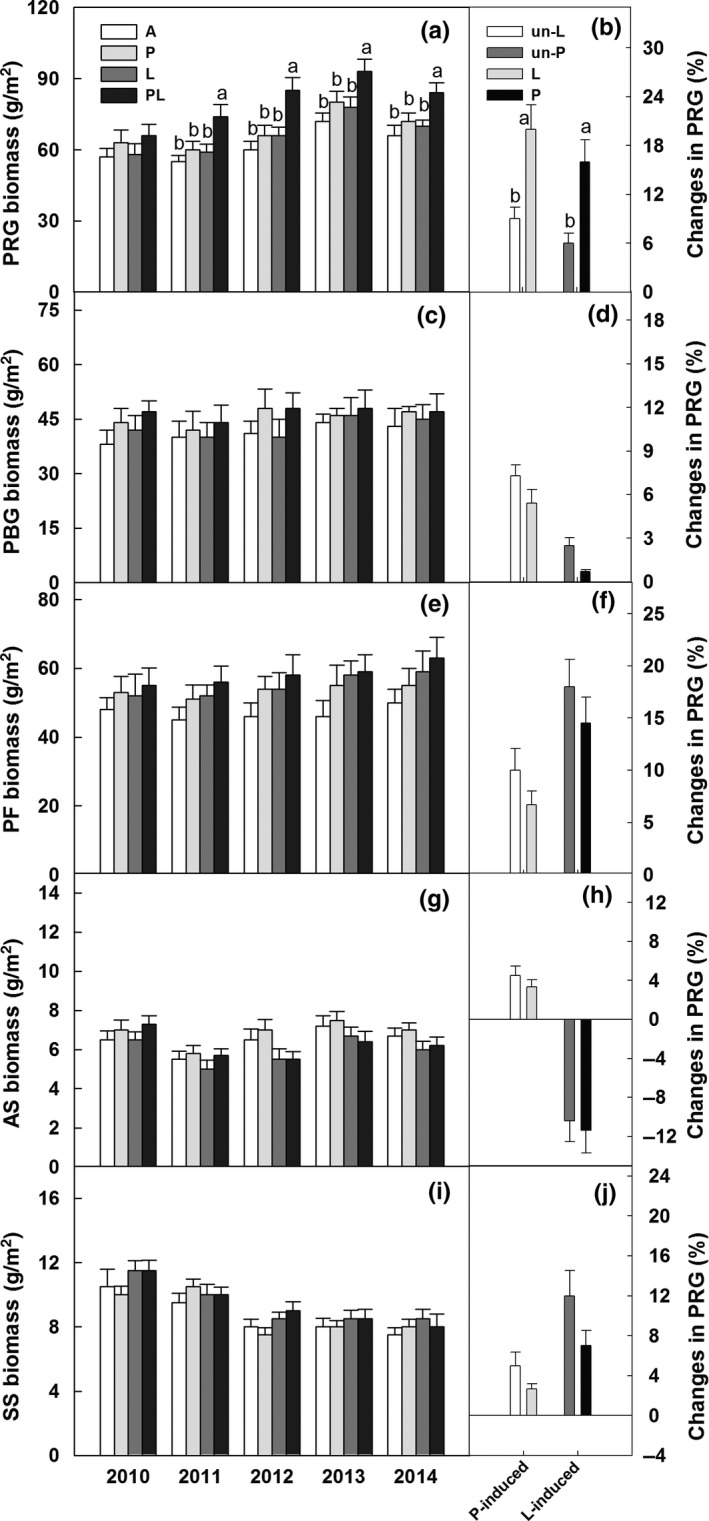
Effects of increased precipitation and belowground litter addition on biomass of perennial rhizome grasses (PRG), perennial bunchgrasses (PBG), perennial forbs (PF), annuals (AS), and semishrubs (SS) from 2010 to 2014 (a, c, e, g, i). Increased precipitation induced changes in biomass of five functional groups in the un‐litter addition plots and litter addition plots, and litter addition induced changes in biomass of five functional groups in the un‐increased precipitation plots and increased precipitation plots after 5 years in the temperate grassland of northeast China (b, d, f, h, j). Vertical bars indicate standard errors of means (*n* = 6). Difference lowercase letters indicate statistically significant differences (*p *<* *.05). A = ambient condition (control), *P* = increased precipitation, *L* = belowground litter addition, PL = increased precipitation and belowground litter addition; un‐L = un‐litter addition, and un‐P = un‐increased precipitation

Plant functional group biomass was significantly different among years (*p *<* *.01; Table 1). However, increased precipitation and belowground litter addition showed no significant effects on biomass of five functional group across the 5 years (Table 1; Figure 4a,c,e,g,i). Significant synergistic interactive effect of increased precipitation and litter addition on PRG was detected (Table 1), because the litter addition induced enhancement of biomass of PRG was significantly higher in the increased precipitation plots than un‐increased precipitation plots after 5 years (Figure 4b,d,f,h,j).

### 3.5 Relationship between plant community and environmental factors

**Figure 5:**
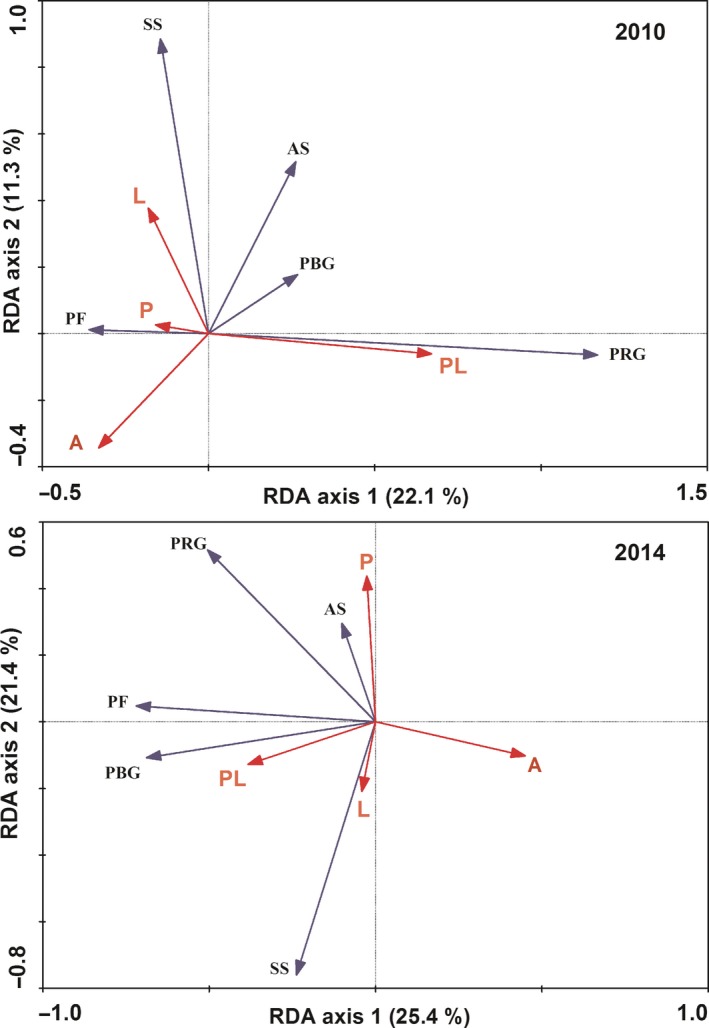
Ordination biplot of redundancy analysis (RDA) displaying the effects of increased precipitation and belowground litter addition on community composition in 2010 and 2014 (a, b). *A* = ambient condition (control), *P* = increased precipitation, *L* = belowground litter addition, PL = increased precipitation and belowground litter addition; abundance of perennial rhizome grasses = abun‐PRG, perennial bunchgrasses = abun‐PBG, perennial forbs = abun‐PF, annuals = abun‐AS, and semishrubs = abun‐SS; biomass of perennial rhizome grasses = bio‐PRG, perennial bunchgrasses = bio‐PBG, perennial forbs = bio‐PF, annuals = bio‐AS, and semishrubs = bio‐SS

Plant community composition was distinguished by treatments with the RDA ordination across the 5 years (Figure 5a,b). In 2010, the first axis explained 22.1% of the variation in plant community composition, mainly associated with increased precipitation and combined increased precipitation and litter addition treatments. The second axis described 11.3% of the variation, primarily related to litter addition treatment. The control, increased precipitation, and litter addition plots had similar community composition (Figure 5a). In 2014, the first axis explained 21.4% of the variation in community composition, mainly related to combined increased precipitation and litter addition treatment. The second axis described 25.4% of the variation, primarily related to increased precipitation and litter addition treatment (Figure 5b).

**Figure 6:**
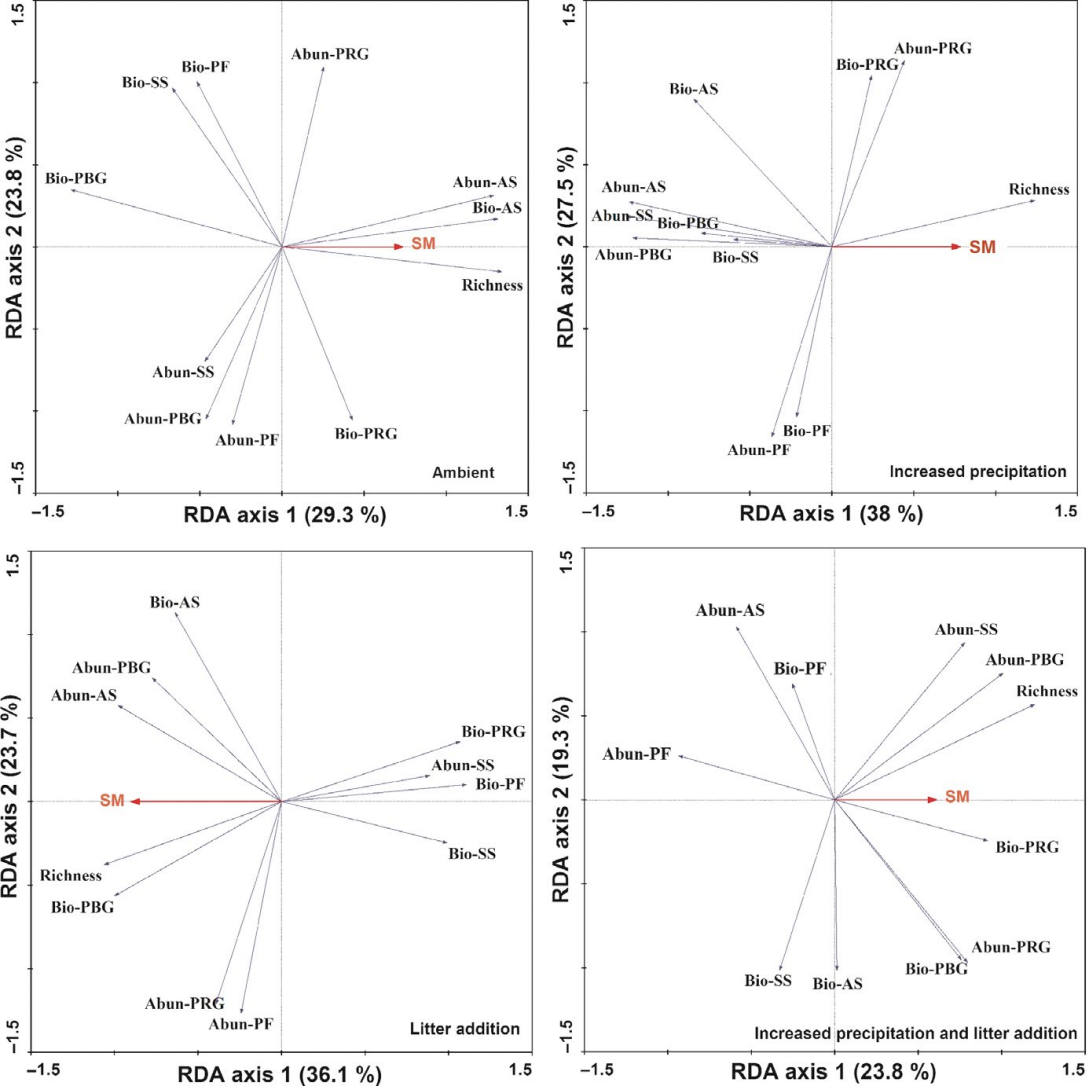
Ordination biplot of redundancy analysis (RDA) displaying the effects of soil moisture on plant species, abundance, and biomass of five functional groups under four treatments across 5 years (a, b, c, d). soil moisture = SM; abundance of perennial rhizome grasses = abun‐PRG, perennial bunchgrasses = abun‐PBG, perennial forbs = abun‐PF, annuals = abun‐AS, and semishrubs = abun‐SS; biomass of perennial rhizome grasses = bio‐PRG, perennial bunchgrasses = bio‐PBG, perennial forbs = bio‐PF, annuals = bio‐AS, and semishrubs = bio‐SS

Stepwise multiple regression analyses were conducted with soil moisture, soil temperature, soil inorganic N, soil organic C, and total N and plant community variables in four treatments across 5 years, and only soil moisture was retained in the models. Redundancy analyses were conducted to represent the relationships among soil moisture and species richness, functional abundance, and biomass of five functional groups (Figure 6). Across the four treatments, the RDA analyses demonstrated plant species richness was positively correlated with soil moisture over the study period. The RDA analyses showed biomass of PRG was negatively correlated with abundance and biomass of PF in the ambient treatments, but it was positively correlated with abundance and biomass of PF under litter addition plots after 5 years (Figure 6; Appendix S3).

## 4 DISCUSSION

### 4.1 Interannual variability in plant community

In temperate ecosystems, one of the most important limiting factor for plant productivity and community composition is soil water availability (Harpole et al., 2007; Hooper & Johnson, 1999); therefore, substantial interannual variation in the measured variables of plant richness, abundance, and biomass of functional groups among the 5 years may be attributed to interannual fluctuations of precipitation in our study. The finding is supported by previous studies in temperate regions which demonstrated that the effects of water addition, N addition, and warming on the abundance and biomass of plant functional groups were dependent of interannual fluctuations of precipitation during the long‐term field experiments (Bai et al., 2004; Bates, Svejcar, Miller, & Angell, 2006; Dukes et al., 2005; Fang, Piao, Tang, Peng, & Ji, 2000; Grime et al., 2000; Hoeppner & Dukes, 2012; Ma et al., 2012; Niu et al., 2009; Yang et al., 2011). For example, a study conducted in the desert steppe has provided evidence that the effects of interannual precipitation would be tremendous and may exceed treatment effects (Hou, Zhou, Xu, Liu, & Zhang, 2013). The treatment effects varied with year in this study suggest that interannual variability of precipitation plays an important impact in regulating the responses of ecosystem structure and function to climatic change.

### 4.2 Increased precipitation effects

The precipitation amount, precipitation frequency, and intensity are all taken into account in this experimental manipulation of increased precipitation treatment. In our study, increased precipitation through iron boxes transferring rains into plots was appropriate than previous studies of water addition treatments which were artificially added water once a week or a month in temperate grasslands (Jin et al., 2010).

In the semiarid temperate grasslands, water availability could affect plant species richness by influencing the establishment and growth of plant species (Niu et al., 2008). The significant increase in plant species richness response to increased precipitation after 5 years is consistent with the studies conducted in other grassland ecosystems (Hou et al., 2013; Robertson, Zak, & Tissue, 2010; Stevens, Dise, & Gowing, 2006; Yang et al., 2011; Zavaleta, Shaw, Chiariello, Mooney, & Field, 2003). RDA and correlation analyses showed that plant species richness was positively correlated with soil moisture across the four treatments (Figure 6; Appendix S3) and thus support the above observation that water availability regulated the responses of plant species richness to environmental changes.

There were consistent positive responses of the abundance of annuals to increased precipitation during the 5 years (Figure 3). This is similar to the observations from some studies in the temperate grassland ecosystems subjected to increased precipitation (Dukes et al., 2005; Zavaleta et al., 2003). For example, Schwinning and Ehleringer (2001) and Yang et al. (2011) demonstrated that shallow‐rooted plants (e.g., annuals) have greater advantage than deep‐rooted plants (e.g., perennial rhizome and bunchgrasses) in acquiring water from shallow soil following precipitation, especially in the water‐limited region. However, we found no effects of precipitation on the biomass of five functional groups during the 5 years (Figure 2). The most probable explanation for the finding is that plant growth may be limited by nutrient availability as precipitation increases in the temperate grasslands.

### 4.3 Belowground litter addition effects

Effects of belowground litter addition on shifts of plant functional group composition were significant after the 5 years. In ambient conditions, abundance and biomass of PRG and PF had strong competition and compensation effect, whereas the competition effect between the two functional groups was reduced with greater amounts of litter (Figures 4 and 5; Appendix 3). The result is inconsistent to previous study in this region, and Wang et al. (2010) have observed that litter addition significantly decreased the competition effect between PBG and PF. Although no certain mechanism could explain this phenomenon, results from different ecosystems indicates that competitive exclusion among different plant functional groups is probably due to nutrient amendment (Gough, Osenberg, Gross, & Collins, 2000).

Reduced plant species richness following the 5 years litter addition in our study is similar to the demonstrated decreases in plant richness with nutrient addition occurring in other ecosystems (Lan & Bai, 2012; Stevens, Dise, Mountford, & Gowing, 2004; Suding et al., 2005). Several mechanisms have been used to explain the reduction in species diversity under litter addition. First, the decrease in species richness under litter litter treatment likely due to altering abiotic conditions (Lu, Mo, Gilliam, Zhou, & Fang, 2010), such as soil moisture (Figure 2). Second, this is likely because the enhanced coverage of PRG and forbs (high‐stature species, data not shown) suppressed growth of annuals (low‐stature species) and suggest that competitive interactions are likely to be strengthened and consequently plant species loss in the temperate grassland in the long term (Yang et al., 2011). Similar to increased precipitation treatment, we also found no effects of litter addition on the biomass of five functional groups during the 5 years (Figure 2). This is likely because plant growth may be limited by water availability as litter addition (i.e., nutrient increase) in our study region.

### 4.4 Interactive effects of increased precipitation and litter addition

There exists significant synergistic interaction between increased precipitation and belowground litter addition on plant biomass, especially biomass of PRG (e.g., *L. chinensis*) (Figure 5). That is, litter addition significantly increased the biomass of PRG under increased precipitation plots, but it showed no effect under ambient precipitation plots during the 5 years. Given the strong response of microbial activity under combined increased precipitation and litter addition treatments (data not shown; Ma et al., 2011), the additions of litter material in our study probably increase soil nutrient availability for plant growth. The positive response of grass biomass to the combined effects could be mainly ascribed to the enhancement of dominant species (e.g., PRG) that can more quickly obtain available water and nutrient resources than other species (Yuan et al., 2005). Our result is consistent to the studies on effects of increased soil nutrient (i.e., N, litter) on plant growth in the semiarid temperate grasslands (Liu et al., 2013; Niu et al., 2009; Wang et al., 2015; Xiao, Janssens, Liu, Zhou, & Sun, 2007). This suggests that plant productivity is nutrient‐limited and highlights the importance of increased precipitation in adjusting the responses of plants to potentially enhanced litter inputs in the semiarid temperate grassland of northeastern China.

Findings from our research indicate that the multifactor effects would be more complex than simple combinations of single‐factor effects. Given expected precipitation regime changes and enhancement of litter inputs under global climatic changes (Schmidt et al., 2011), multifactor field experiments are expected to gain further insight into the impacts of global climatic changes on terrestrial ecosystem structure and function.

## 5 CONCLUSIONS

With a 5‐year field manipulative experiment, this study showed that increased precipitation significantly increased plant species richness and abundance of annuals, whereas litter addition decreased them. PRG and forbs had strong competition effect under ambient levels, whereas the competition between the two functional groups disappeared under litter addition plots. In addition, combined increased precipitation and litter addition caused significant interactive effects on community composition, because litter addition significantly increased biomass and abundance of rhizome grasses under increased precipitation plots but showed no effect under ambient precipitation levels. Our findings highlight that water availability would regulate the effects of potentially enhanced litter inputs in the semiarid temperate grasslands. Further long‐term multifactorial field experiments will be needed to capture potential effects of global climatic changes on plant community composition and structure.

## CONFLICT OF INTEREST

None declared.

## AUTHOR CONTRIBUTIONS

Renzhong Wang conceived and designed the experiments. Linna Ma wrote the main manuscript text and analyzed the data. Hongxia Chen performed the experiments and processed the data. Xiaoping Xin and Junyao Liu performed the experiments. All authors reviewed the manuscript.

## DATA ACCESSIBILITY

All data are included in the manuscript and Support information.
