# Supplementary material for: Plant community responses to increased precipitation and belowground litter addition: Evidence from a 5‐year semiarid grassland experiment
Source: Ecol Evol. 2018 Apr 14;8(9):4587–97. doi: 10.1002/ece3.4012 (PMC5938451; doi:10.1002/ece3.4012)
Supplement: Supplementary file 1 [file ECE3-8-4587-s001.docx]

**Support information**

**Appendix 1.** The picture of increased precipitation treatments in a semi-arid temperate grassland of northeast China.

**Appendix 2.** The plant species, life form (LF), life history type (LHT) and the percent

cover of plant species within the quadrats (1 × 1 m). Perennial rhizome grasses (PRG),

perennial bunchgrasses (PBG), perennial forbs (PF), annuals (AS) and semi-shrubs (SS).

| **Species** | **LF** | **LHT** | **Percent cover %** |
| --- | --- | --- | --- |
| *Leymus chinensis* | Grass | PRG | 15 |
| *Carex tristachya* | Grass | PRG | 5 |
| *Stipa capillata* | Grass | PBG | 10 |
| *Achnatherum sibiricum* | Grass | PBG | 1 |
| *Cleistogenes squarrosa* | Grass | PBG | 1 |
| *Poa annua* | Grass | PBG | 1 |
| *Artemisia dracunculus* | Forb | PF | 5 |
| *Artemisia tanacetifolia* | Forb | PF | 4 |
| *Serratula centauroides* | Forb | PF | 3 |
| *Pulsatilla chinensis* | Forb | PF | 2 |
| *Thalictrum squarrosum* | Forb | PF | 3 |
| *Allium tenuissimum* | Forb | PF | 2 |
| *Potentilla acaulis* | Forb | PF | 2 |
| *Potentilla verticillaris* | Forb | PF | 1 |
| *Potentilla bifurca* | Forb | PF | 2 |
| *Orostachys fimbriatus* | Forb | PF | 1 |
| *Vicia amoena* | Forb | PF | 1 |
| *Astragalus angustifoliolatus* | Forb | PF | 1 |
| *Adenophora stricta* | Forb | PF | 1 |
| *Bupleurum chinensis* | Forb | PF | 2 |
| *Galium verum* | Forb | PF | 1 |
| *Thermopsis lanceolala* | Forb | PF | 1 |
| *Scorzonera austriaca* | Forb | PF | 1 |
| *Carex pediformis* | Forb | PF | 4 |
| *Iris ventricosa* | Forb | PF | 2 |
| *Heteropappus altaicus* | Forb | PF | 1 |
| *Tephroseris kirilowii* | Forb | PF | 1 |
| *Lychnis fulgens* | Forb | PF | 1 |
| *Astragalus membranaceus* | Forb | PF | 1 |
| *Dontostemon micranthus* | Annual | AS | 2 |
| *Artemisia frigida* | Semi-shrub | SS | 2 |
| *Lespedeza bicolor* | Semi-shrub | SS | 1 |

**Appendix 3.** Correlation coefficients among soil moisture (SM), plant species richness (PSR), abundance of perennial rhizome grasses (A-PRG), perennial bunchgrasses (A-PBG), perennial forbs (A-PF), annuals (A-AS) and semi-shrubs (A-SS), and biomass of perennial rhizome grasses (B-PRG), perennial bunchgrasses (B-PBG), perennial forbs (B-PF), annuals (B-AS) and semi-shrubs (B-SS) under ambient, increased precipitation (P), belowground litter addition (L) and combined increased precipitation and litter addition treatments.

| ***Ambient*** | | | | | |  | |  | |  |  |  |  |  |  |  |
| --- | --- | --- | --- | --- | --- | --- | --- | --- | --- | --- | --- | --- | --- | --- | --- | --- |
|  | | | **SM** | **PSR** | | **A-PRG** | | **A-PBG** | | **A-PF** | **A-AS** | **A-SS** | **B-PRG** | **B-PBG** | **B-PF** | **B-AS** |
| **PSR** | | | **0.885^*^** |  | |  | |  | |  |  |  |  |  |  |  |
| **A-PRG** | | | 0.179 | 0.034 | |  | |  | |  |  |  |  |  |  |  |
| **A-PBG** | | | -0.758 | -0.658 | | -0.775 | |  | |  |  |  |  |  |  |  |
| **A-PF** | | | -0.237 | -0.084 | | **-0.912^**^** | | 0.801 | |  |  |  |  |  |  |  |
| **A-AS** | | | **0.851^*^** | 0.884 | | 0.438 | | -0.889 | | -0.505 |  |  |  |  |  |  |
| **A-SS** | | | -0.353 | -0.193 | | -0.663 | | 0.722 | | 0.749 | -0.605 |  |  |  |  |  |
| **B-PRG** | | | 0.320 | 0.445 | | -0.873 | | 0.376 | | 0.830 | 0.055 | 0.580 |  |  |  |  |
| **B-PBG** | | | -0.946 | **-0.886*** | | 0.135 | | 0.522 | | -0.084 | -0.802 | 0.080 | -0.589 |  |  |  |
| **B-PF** | | | -0.371 | -0.480 | | 0.830 | | -0.322 | | -0.773 | -0.130 | -0.298 | **-0.901**** | 0.616 |  |  |
| **B-AS** | | | **0.869^*^** | 0.914 | | 0.316 | | -0.816 | | -0.392 | 0.690 | -0.574 | 0.184 | -0.853 | -0.263 |  |
| **B-SS** | | | -0.489 | -0.616 | | 0.766 | | -0.642 | | -0.732 | -0.214 | -0.426 | -0.970 | 0.739 | 0.954 | -0.327 |
|  | | |  |  | |  | |  | |  |  |  |  |  |  |  |
| ***Increased precipitation*** | | | | | |  | |  | |  |  |  |  |  |  |  |
|  | | **SM** | | **PSR** | | **A-PRG** | | **A-PBG** | | **A-PF** | **A-AS** | **A-SS** | **B-PRG** | **B-PBG** | **B-PF** | **B-AS** |
| **PSR** | | **0.878*** | |  | |  | |  | |  |  |  |  |  |  |  |
| **A-PRG** | | 0.337 | | 0.527 | |  | |  | |  |  |  |  |  |  |  |
| **A-PBG** | | **-0.855*** | | -0.916 | | -0.230 | |  | |  |  |  |  |  |  |  |
| **A-PF** | | -0.303 | | -0.497 | | **-0.829**** | | 0.205 | |  |  |  |  |  |  |  |
| **A-AS** | | **-0.875*** | | -0.906 | | -0.119 | | 0.758 | | 0.084 |  |  |  |  |  |  |
| **A-SS** | | **-0.867*** | | -0.934 | | -0.191 | | 0.671 | | 0.157 | **0.865**** |  |  |  |  |  |
| **B-PRG** | | 0.181 | | 0.337 | | 0.744 | | -0.288 | | -0.767 | -0.027 | -0.103 |  |  |  |  |
| **B-PBG** | | -0.625 | | -0.573 | | 0.000 | | 0.829 | | 0.000 | 0.673 | 0.688 | -0.447 |  |  |  |
| **B-PF** | | -0.181 | | -0.337 | | -0.744 | | 0.288 | | 0.767 | 0.027 | 0.103 | **-0.896*** | 0.447 |  |  |
| **B-AS** | | -0.652 | | -0.478 | | 0.494 | | 0.703 | | -0.524 | 0.805 | 0.759 | 0.417 | 0.594 | -0.417 |  |
| **B-SS** | | -0.474 | | -0.427 | | 0.048 | | 0.713 | | -0.040 | 0.527 | 0.544 | -0.491 | 0.983 | 0.491 | 0.495 |
|  | |  | |  | |  | |  | |  |  |  |  |  |  |  |
| ***Litter addition*** | | | | | |  | |  | |  |  |  |  |  |  |  |
|  | | **SM** | | | **PSR** | **A-PRG** | | **A-PBG** | | **A-PF** | **A-AS** | **A-SS** | **B-PRG** | **B-PBG** | **B-PF** | **B-AS** |
| **PSR** | | **0.754*** | | |  |  | |  | |  |  |  |  |  |  |  |
| **A-PRG** | | 0.364 | | | 0.626 |  | |  | |  |  |  |  |  |  |  |
| **A-PBG** | | 0.690 | | | 0.571 | -0.023 | |  | |  |  |  |  |  |  |  |
| **A-PF** | | 0.227 | | | 0.508 | **0.890*** | | -0.129 | |  |  |  |  |  |  |  |
| **A-AS** | | 0.774 | | | 0.742 | 0.030 | | 0.742 | | -0.103 |  |  |  |  |  |  |
| **A-SS** | | -0.797 | | | -0.719 | -0.161 | | -0.202 | | -0.046 | -0.519 |  |  |  |  |  |
| **B-PRG** | | **-0.866*** | | | **-0.877*** | -0.524 | | -0.491 | | -0.400 | -0.718 | 0.851* |  |  |  |  |
| **B-PBG** | | 0.898 | | | 0.588 | 0.736 | | 0.491 | | 0.632 | 0.649 | -0.655 | -0.649 |  |  |  |
| **B-PF** | | **-0.808**** | | | **-0.872*** | -0.426 | | -0.669 | | -0.293 | -0.851 | 0.786** | **0.776*** | -0.626 |  |  |
| **B-AS** | | 0.567 | | | 0.299 | -0.549 | | 0.527 | | -0.660 | 0.601 | -0.478 | -0.370 | 0.153 | -0.511 |  |
| **B-SS** | | -0.697 | | | -0.561 | -0.031 | | -0.509 | | 0.105 | -0.767 | 0.540 | 0.563 | -0.666 | 0.673 | -0.721 |
|  | |  | | |  |  | |  | |  |  |  |  |  |  |  |
| ***Increased precipitation and litter addition*** | | | | | | | | | | |  |  |  |  |  |  |
|  | **SM** | | | **PSR** | | **A-PRG** | **A-PBG** | | **A-PF** | | **A-AS** | **A-SS** | **B-PRG** | **B-PBG** | **B-PF** | **B-AS** |
| **PSR** | **0.733*** | | |  | |  |  | |  | |  |  |  |  |  |  |
| **A-PRG** | 0.497 | | | -0.072 | |  |  | |  | |  |  |  |  |  |  |
| **A-PBG** | 0.607 | | | 0.629 | | -0.308 |  | |  | |  |  |  |  |  |  |
| **A-PF** | -0.348 | | | 0.245 | | **-0.904*** | 0.422 | |  | |  |  |  |  |  |  |
| **A-AS** | **-0.753*** | | | -0.127 | | -0.639 | 0.639 | | 0.612 | |  |  |  |  |  |  |
| **A-SS** | 0.461 | | | 0.676 | | -0.519 | 0.664 | | 0.632 | | 0.645 |  |  |  |  |  |
| **B-PRG** | 0.564 | | | 0.649 | | 0.496 | -0.124 | | -0.378 | | -0.834 | -0.120 |  |  |  |  |
| **B-PBG** | 0.469 | | | 0.000 | | **0.811*** | -0.392 | | -0.556 | | -0.776 | -0.567 | 0.632 |  |  |  |
| **B-PF** | -0.664 | | | -0.649 | | -0.496 | 0.124 | | 0.378 | | 0.834 | 0.120 | -0.362 | -0.632 |  |  |
| **B-AS** | 0.006 | | | -0.651 | | 0.778 | -0.456 | | -0.866 | | -0.244 | -0.672 | -0.130 | 0.684 | 0.130 |  |
| **B-SS** | -0.206 | | | -0.462 | | 0.696 | -0.895 | | -0.768 | | -0.813 | -0.958 | 0.374 | 0.761 | -0.374 | 0.670 |

** and ** represent significant at P < 0.05 and 0.01, respectively.*
